# Epidemiological investigations in a digital era: methods and tools to identify infectious disease outbreaks

**DOI:** 10.1186/2047-2994-4-S1-P231

**Published:** 2015-06-16

**Authors:** T Braun, J Tarabeia, V Schechner, Y Carmeli

**Affiliations:** 1 Epidemiology, Tel Aviv Sourasky Medical Center, Israel

## Introduction

Outbreak investigations are time-consuming. Containing an outbreak requires quick analysis and response. At the Tel Aviv Sourasky Medical Center Epidemiology Department, we developed methods and tools based on several computerized algorithms to identify infectious diseases outbreaks and assist in their investigation.

## Objectives

To describe the methods and share the tools we developed for outbreak identification and investigation.

## Methods

We use databases that contain data gathered from various systems in the hospital and then integrate, clean and reorganize them in an optimized way for advanced analysis. We developed algorithms that scan the databases and automatically identify outbreaks; then, queries alert us when an outbreak occurs. Additional queries detect the index patient and identify patients and staff who were exposed to the index and require screening or isolation.

## Results

Compared to the non-automated methods used earlier, the new system has identified nearly 3 times the number of contacts who require screening. Moreover, because we are able to identify and isolate the index case more quickly, the number of positive contacts per index case has declined by half. The system has shortened the time needed for each investigation from several days to a few hours.

## Conclusion

Tools to assist with outbreak investigations can be developed relatively easily building upon existing databases in the hospital. Such tools improve the efficiency and accuracy of the investigations, and ultimately lead to reduction in transmission.

## Disclosure of interest

None declared.

